# Biological sex estimation in experimentally burnt patellae: exploring sexual dimorphism through comparative analysis

**DOI:** 10.1007/s00414-025-03467-5

**Published:** 2025-03-14

**Authors:** Beatriz Mouga Almeida, Filipa Cortesão Silva, Ana Luísa Santos

**Affiliations:** 1 Laboratory of Forensic Anthropology, Department of Life Sciences, University of Coimbra, Calçada Martim de Freitas, Coimbra, 3000-456 Portugal; 2 Department of Prehistory and Archaeology, University of Seville, C/ Doña María de Padilla, s/n, Seville, 41004 Spain; 3 Research Centre for Anthropology and Health (CIAS), Department of Life Sciences, University of Coimbra, Rua do Arco da Traição, Coimbra, 3000-056 Portugal; 4 Centre for Functional Ecology, Department of Life Sciences, University of Coimbra, Calçada Martim de Freitas, Coimbra, 3000-456 Portugal

**Keywords:** Biological profile, Metric method, Thermally induced changes, Cremation

## Abstract

The patella has been used in various studies to verify its value in the estimation of biological sex. However, there is limited understanding regarding the alterations the bone undergoes when exposed to the effects of fire and how it affects sexual dimorphism. The current study aims to study the efficacy of three patella measurements, and generate an equation and cut-off points, to estimate the sex of individuals that had their patellae subjected to burning. Furthermore, the applicability of cut-off points from two previous studies was tested. Patellae (*n* = 32 individuals) from the sub-collection of experimentally burned skeletons at the 21st Century Identified Skeletal Collection were measured with a digital calliper for their maximum height, maximum thickness, and maximum width. The sample comprises 18 females (56.2%) and 14 males (43.8%) with ages at death between 60 and 93 years (x̄ = 78.6 years). The measurements were evaluated through linear discriminant analysis for sex estimation allowing correct classifications between 68.8 and 75%. The sexual dimorphism in both burnt and unburnt patellae was studied and results were significant for maximum height and maximum width. It was concluded that sex estimation is possible in patellae that have been subjected to different degrees of burning and sexual dimorphism is maintained albeit at lower levels compared to the non-burnt patellae. This study brought light into use of experimentally burnt patellae with its possible implications for forensic investigations although further studies with larger sample sizes are needed.

## Introduction

Sex estimation is a fundamental step in the process of identifying human remains, especially in Forensic Anthropology [1], as it is one of the four primary components of the biological profile [2]. This estimation is typically performed by analysing various skeletal traits [3], which display sexual dimorphism in humans [4]. It is critical to be aware that sex estimation is based on the evaluation of an individual’s biological sex based on skeletal markers and therefore it is different from gender, which is based on an individual’s self-reported identity [5]. While gender identity is a complex and multifaceted concept that can vary across cultures and individuals [6], biological sex is determined by the presence of specific biological and physical characteristics [3, 7]. Thus, sex estimation focuses on identifying these biological traits in skeletal remains to make an accurate estimation of the individual’s sex [2, 8]. However, and as Geller [9] has stated, the skeletal analysis to estimate the biological sex of an individual is not entirely a dichotomous and unchanged parameter.

The patella is part of the knee joint and is protected due to the presence of the quadriceps tendon [10]. During the body initial state of decomposition, it is sheltered from external forces and less susceptible to taphonomic changes, such as weathering [11–14].

The use of the patella for biological sex estimation in different populations [1, 11, 15–19] has been documented in several studies, which have demonstrated its potential to provide valuable information on the sex of individuals, particularly in situations where more dimorphic bones (e.g. pelvis or skull [7]) are unavailable or poorly preserved.

Maio and colleagues [18] developed in the 21st Century Identified Skeletal Collection (ISC/XXI) at the University of Coimbra a study where the patella was confirmed as a good bone for sex estimation in the Portuguese population, reaching values of 80.2% of accuracy after cross validation. As seen in previous studies [11, 16, 17, 20], the measurements that render the highest accuracy in sex estimation are patellar maximum height, thickness and width. This was then corroborated in ISC/XXI [18].

Thermally changed bones can pose a challenge in sex estimation. However, various studies have shown that bones with these changes can still exhibit sexual dimorphism [21–30]. The morphological and metric methods commonly used for sex estimation in the unburnt bone can also be applied to the calcined bone although some modifications may be necessary due to changes in bone structure caused by heat [23, 25, 31]. It is important to note that the accuracy of sex estimation in calcined bone may be affected by the degree of burning and other factors such as fragmentation [23, 25, 30, 32, 33], making it crucial to approach such cases with caution and to consider multiple indicators for sex estimation. Concerning the patella, it can be most affected by the pugilistic stance, also known as the “boxer’s pose,” which occurs when a body is exposed to intense heat, causing the muscles to contract due to protein denaturation and rapid dehydration [30–32]. This results in the limbs flexing tightly, with the arms and legs bending at the elbows and knees, and the hands clenching into fists [32, 33]. Moreover, the patella can be damaged first because the knee joint is prominent when the flexing of the lower limbs occurs, thus more affected throughout the heat exposure [31, 33]. However, it is not unusual to find the patella, although not always in a complete state, as well as other small bones that have similar amounts of trabecular bone such as the carpals and tarsals, from archaeological and commercial cremations. This is seen in previous research, such as Cavazutti and colleagues [34], where the patellae are frequently found for full evaluation in ancient cremations. In the case of modern commercial cremations, Hlad et al. [35] shows that the patellae are one of the most fully preserved bones, particularly for the maximum thickness. Thus, it could be extrapolated to forensic contexts.

Up until this point, from the author’s knowledge, only two known studies [34, 35] have employed the patella with heat-induced alterations to created cut-off points and analysed sexual dimorphism for the three most dimorphic measurements of the patella– maximum height, thickness, and width. Cavazzuti and colleagues [34] carried out the measurements on the skeletal remains of 124 individuals, from the Late Bronze Age and Iron Age. The individuals were adults whose biological sex was inferred from gendered grave goods. The study highlights the potential of the patella bone as a reliable source of information for sex estimation in cremated remains. Note that in archaeological contexts, when looking at cremation graves sex estimation can only be done in around 50% of individuals [34, 35]. This value is explained due to the skeleton only being partially represented in cremation scenarios, in most cases, as well as bones have undergone changes through shrinking, deformation and fragmentation [36, 37]. Due to this, various bones cannot be evaluated through standard morphological and metric methods for biological sex estimation [37]. Similarly, Hlad and researchers [35] used a reference collection of 87 adult cremated individuals with known sex and age at death from the William M. Bass Donated Skeletal Collection in Knoxville, USA. The study suggests similar conclusions to the previously mentioned [34], where patellar measurements are a useful tool for sex estimation in burned human remains and can provide accurate results when combined with machine learning algorithms. However, differences in the cremation process between the two studies need to be considered, as the modern collection had the individuals completely cremated through the rules of USA crematoria in a controlled environment, whereas the Italian sample was burned, most likely, in a traditional pyre subject to environmental factors and relied on bone colouring and condition to infer complete calcination.

The goal of the current study is to take advantage of the pioneer and unique, sub-collection of experimentally burnt skeletons, part of the 21st Century Identified Skeletal Collection, and develop a methodology that allows to do the sex estimation using the patella, of thermally altered skeletal remains. This is to be achieved by calculating the sexual dimorphism in the burnt and unburnt patella, to observe the differences and through linear discriminant analysis.

Furthermore, this is a reliable bone for sex estimation in non-burnt individuals in the Portuguese population, so it is beneficial to create a similar method for burned bones. Additionally, a comparison is to be made with the results obtained by two previous studies [34, 35], to gauge the differences between studies.

## Materials and methods

The left (unburnt) and right (calcined) patellae from 32 individuals’ part of the sub-collection of Identified Burned Skeletons at the 21st Century Identified Skeletal Collection (ISC/XXI), which is located in the Laboratory of Forensic Anthropology at the University of Coimbra [38], were measured. This sub-collection consists of a total of 56 individuals [38], however, due to alterations that occurred during the burning process, 24 burnt patellae had their measurement rendered impossible. Note that no individual that has pathological alterations or substantial entheseal changes are chosen to burn [38], and the authors did not measure any patellae with major changes that avoid a correct measurement, such as heat induced fracturing. Measurements were taken previously in 30 individuals, where the patellae had not been subjected to prior burning, in order to grasp the needed technique. Each burnt patella was measured only once due to the fragility of the samples. The burning procedure occurred in the context of previous research, with the right antimere of paired bones being burnt [39]. The patellae were subjected to heat, under controlled conditions, using an electric muffle furnace (Barracha, K-3 three-phased) and the temperature measured through a type K probe, adhering to the norm IEC 60584-2 [38, 39]. Particular data pertaining to the duration of burning and temperature is available for each skeleton upon request, with the duration range going from 90 to 240 min (average time span of 165 min) and temperature ranging from 450 to 1100º C (average temperature 775ºC) [39]. The patellae were analysed and considered calcined when they had the characteristic white porcelain colour, in accordance with Shipman et al. (1984).

The patellae sample in this study comprises 18 females (56.2%) with ages at death between 62 and 92 years old (x̄ = 80.1, s.d = 5.86) and 14 males (43.8%) with an age at death between 60 and 93 years old (x̄ = 76.6, s.d. = 10.5). Three measurements, maximum height (MAXH), maximum thickness (MAXT) and maximum width (MAXW) (Table 1 [1^2^]), were taken using a digital calliper with a precision of 0.01 mm in both patellae from the same individual. These measurements were chosen as they are the most dimorphic according to previous studies [11,34,35].

**Table 1:** Patella measurement descriptions. (adapted from Introna and colleagues [11])

Measurement	Description	
Maximum height (MAXH)		Maximum distance between the base and the vertex
Maximum Thickness (MAXT)		Maximum distance between the anterior and posterior facet
Maximum width (MAXW)		Maximum distance between the lateral and medial extremes

To minimize the handling of burned bones, the intra- and inter-observer error was calculated by using 30 individuals, to measure both right and left patellae, from the Coimbra Identified Skeletal Collection, as the burnt patella were too fragile. This allowed confidence in the measurements to be taken in the burnt patellae. The calculations used were technical error of measurement (TEM), the relative technical error of measurement (%TEM), as well as the coefficient of reliability (R) [40]. The values of TEM (from 0.30 intraobserver error to 0.48 interobserver error) and %TEM (between 0.74 intraobserver error to 2.50 interobserver error) were relatively low for both right and left patellae, which indicates good quality of measurements that were not significantly affected by systematic errors. The coefficients of reliability were all very close to 1 (from 0.97 to 0.99), showing a high-level consistency within the study.

The dataset obtained for the experimentally burnt patellae was used and an initial descriptive analysis for each measurement was made to obtain the means and standard deviations. To verify if the data follows a normal distribution a Shapiro-Wilk test was made for each measurement [41]. A Levene’s test was employed to assess the equality of variances among the groups and to examine whether there was a significant difference in the variability of measurements [42]. To gauge the size differences between patellae from females and males, Mann-Whitney U tests were run for each measurement. This statistical test was chosen as it determines if there are statistical differences in the means of the two groups, therefore if there are differences in the size, assuming independent observations [43].

To further analyse the sexual dimorphism of each measurement, the calculation of cut-off points and the determination of the D-value [44] were employed. The cut-off point was calculated based on the means and standard deviations of the measurements taken for males and females, using Python (following the procedures used by Cavazzuti and colleagues [34]). When this value is obtained it allows the classification of the individual as female, if their measurement falls below the value, whereas if the value is higher the individual is classified as male. The formula employed was:

$$\:D-value=F\:\left({x}_{0}\right|\:{x}_{f},\:{\:S}_{f}^{2})-F\:({x}_{0}|\:{x}_{m},\:{\:S}_{m}^{2})$$

Where the cumulative distribution function is denoted by F (x), x0 the cut-off point, xf the mean of the female distribution, xm the mean of the male distribution, sf the variance of females and sm the variance of males [45]. To be able to visualize the degree of sexual dimorphism within the measurements, overlapping histograms with their respective normal distribution curves were created. The same calculations were made to the left unburnt patellae data to verify how sexual dimorphism varied in the sample. To check the accuracy of the cut-off points in the non-burned patellae, the measurements of a randomized group of 30 individuals from ISC was chosen, with an equal division of sexes.

Subsequently, a linear discriminant analysis (LDA) was performed on the burnt antimere values to construct a predictive model for determining the most probable group - male or female - to which a patella belongs. Cross-validation is performed within the sample in linear discriminant analysis to assess the performance of the classification model and to estimate how well it can predict the sex of an individual in new samples [18, 19]. Thereafter, the cut-off points obtained from previous studies [34, 35] were assessed for their applicability in the experimentally burnt sample to determine their reliability in these individuals under analysis. This was done by comparing the cut-off points to the data of the calcined patellae, as the studies were developed on calcined bone samples. Therefore, the patellae that were subjected to temperatures equal or over 800ºC and displayed a white colour were chosen. Statistical tests were performed by using the software IBM SPSS ^®^ version 29.0.0 and Python Programming Language.

## Results

**Fig. 1:**
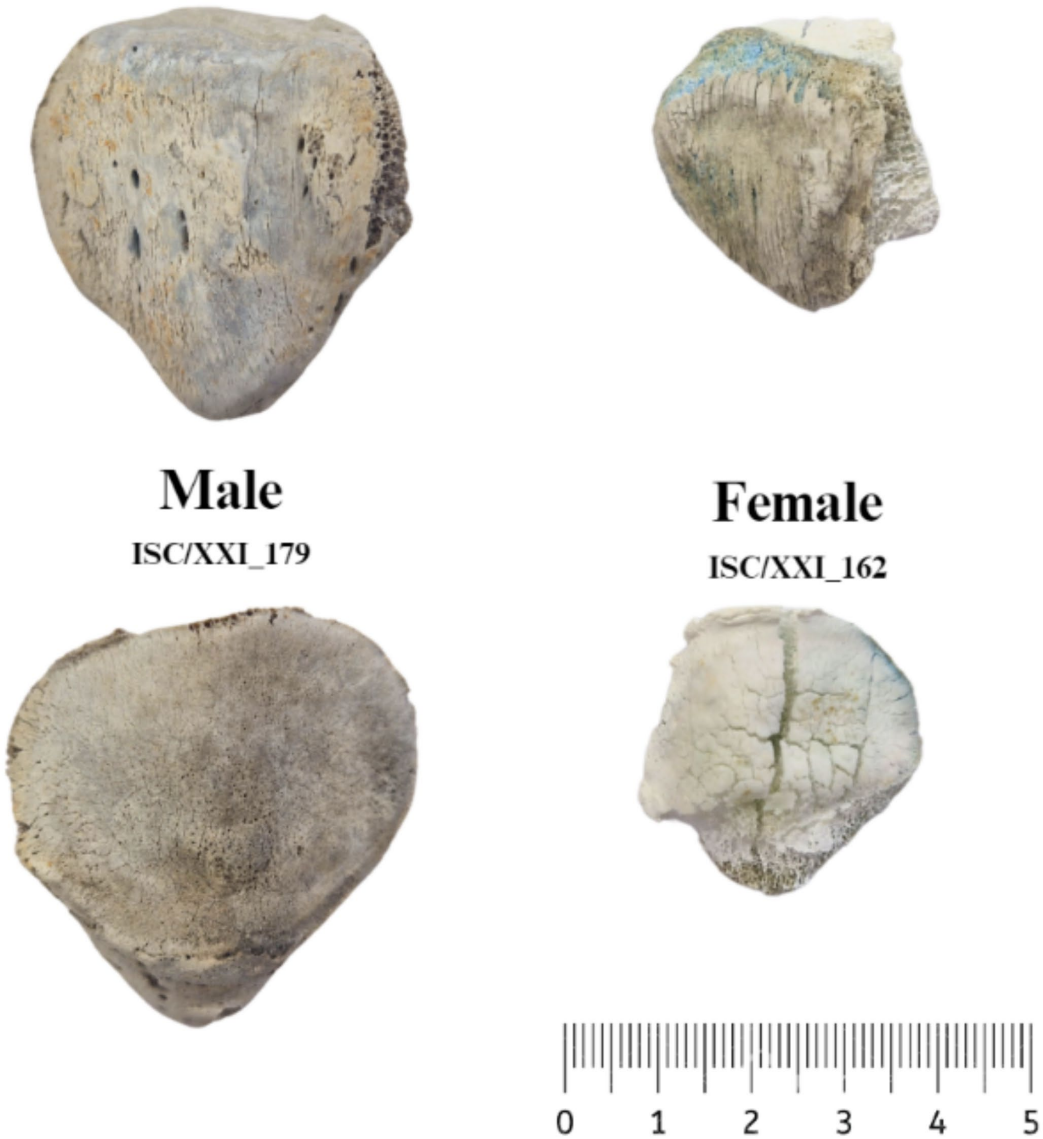
Representation of the male and female patellae, burned at two different degrees of combustion. ISC/XXI_179 at 700ºC and 90 min and ISC/XXI_162 at 1100ºC and 120 min

The distribution of measurements was analysed to determine their normality, and it was found that all groups had a low value of skewness (ϒ < 1), suggesting a non-normal distribution. The Shapiro-Wilk test corroborated this assumption, as the *P*-values obtained were higher than the threshold of 0.05, which indicates that the data significantly deviates from a normal distribution. To test for homogeneity of variance, a Levene’s test was conducted, which showed no statistical significance (*p* > 0.05). A representation of the variety and burning degrees seen in the sample is given in Fig. 1.

Mann-Whitney U test was performed to assess the differences in sexual dimorphism between measurements, revealing significant differences (*sig*. > 0.05) for two measurements, MAXH and MAXW, while MAXT showed no significant difference (Table 2). D-value are low in each of the measurements, all lower than 0.5, with the MAXW (0.453) having the highest value. The D-values and cut-off points were also calculated for the left unburnt patellae (Table 3), with the D-values being higher than in the burnt patellae.

**Table 2:** Descriptive statistics, D-values and cut-off points obtained for each of the measurements in the experimentally burnt patellae, the measurements were taken in the patellae of 18 females and 14 males

Measurement	Females				Males				Test stat	Sig.	D value	Cut- off point (mm)
	mean (mm)	s.d. (mm)	min (mm)	max (mm)	mean (mm)	s.d. (mm)	min (mm)	max (mm)				
MAXH	33.33	4.53	27.11	42.93	38.22	4.23	31.42	25.49	-2.70	0.007	0.423	35.87
MAXT	17.01	1.96	14.48	21.82	18.26	2.49	14.60	23.32	-1.25	0.210	0.220	17.55
MAXW	34.90	4.43	28.33	44.96	39.58	3.35	32.32	44.24	-2.81	0.005	0.453	37.53

MAXH– maximum height; MAXT– maximum thickness; MAXW– maximum width; s.d.– standard deviation; Sig.– statistical significance; D value [44]

The cut-off points pertaining to the non-burnt patellae (Table 3) were tested and it was verified that all measurements had a correct classification equal or greater than 80%. MAXH achieved the highest results, particularly for females, with an 87% correct classification rate.

**Table 3:** Descriptive statistics, D-values and cut-off points obtained for each of the measurements in the unburnt antimere patellae, the measurements were taken in the patellae of 18 females and 14 males

Measurement	Females				Males				Test stat	Sig.	D value	Cut- off point (mm)
	mean (mm)	s.d. (mm)	min (mm)	max (mm)	mean (mm)	s.d. (mm)	min (mm)	max (mm)				
MAXH	37.95	2.98	30.17	33.48	42.45	2.23	39.66	46.38	3.38	< 0.001	0.019	40.20
MAXT	18.83	1.82	16.08	22.00	20.52	1.39	17.54	22.37	2.96	0.002	0.587	19.41
MAXW	38.99	3.26	44.39	45.95	43.81	2.53	40.74	49.13	3.31	< 0.001	0.785	41.28

MAXH– maximum height; MAXT– maximum thickness; MAXW– maximum width; s.d.– standard deviation; Sig.– statistical significance; D value [44]

The results of the linear discriminant analysis (Table 4) indicate that the accuracy of correctly classifying the sex of the sample is higher for females than for males. MAXW is the measurement with the highest percentage of correct classification (75.0%), after cross-validation. Before cross-validation, the combination of the three measurements provided the most accurate classification with a rate of 78.8%, but this value was reduced to 71.9% after cross-validation was employed. The centroid values obtained for each trait and the combination of the three also demonstrate their accuracy in sex classification. The combined measurement’s function displayed the highest separation between males and females with centroid values of 0.732 and − 0.569, respectively, indicating the most precise function for estimating sex. The results of the linear discriminant analysis conducted in this study indicate that the three measurements analysed have a high correct percentage rate for estimating sex in the patellae of burnt individuals.

**Table 4:** Linear discriminant analysis results with the correct classification percentage, centroid values and coefficients obtained for each of the measurements and the combined function

Function	Measurement	Unstandardized Coefficients	Centroids		Classification (%)	
			Females	Males	O	CV
1	MAXH	0.277	-0.485	0.624	71.9	71.9
	Constant	-8.052				
2	MAXT	0.444	-0.242	0.312	71.9	68.8
	Constant	-7.791				
3	MAXW	0.247	-0.507	0.652	75.0	75.0
	Constant	-9.140				
4	MAXH	0.159	-0.569	0.732	78.1	71.9
	MAXT	-0.302				
	MAXW	0.193				
	Constant	-7.448				

O– original; CV– cross-validation

Based on the higher correct classification percentage value, the following equation is optimized to use within the specific sample:

$$\:\varvec{Y}=\left(\varvec{M}\varvec{A}\varvec{X}\varvec{H}\:\times\:0.159\right)+\left(\varvec{M}\varvec{A}\varvec{X}\varvec{T}\:\times\:\:-0.302\right)$$

$$\:+\left(\varvec{M}\varvec{A}\varvec{X}\varvec{B}\:\times\:0.193\right)-7.448$$

**Fig. 2:**
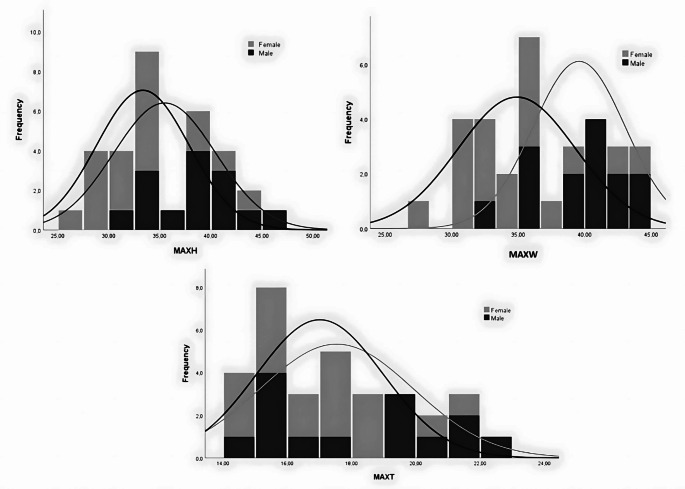
Q1

The point of intersection of the two normal distribution lines represents the cut-off point of each measurement, as seen in Fig. 2, where the area of non-overlap between the two distributions entails the D- value. MAXT and MAXW display the least amount of overlap, indicating that they are the most effective traits for sex estimation in the study sample.

The D-values and cut-off points obtained for the calcined specimens were then calculated (Table 5), as well as the linear discriminant analysis classification (Table 5), for each of the patella measurements to compare the cut-off points of the chosen studies [34, 35].

**Table 5:** D-values, cut-off points and correct classification percentage obtained for each of the three measurements in the calcined patellae: 13 from females, and 10 from males. Total sample of 23 patellae pertains to the calcined only

Measurement	D-value	Cut-off point	LDA %correct classification
MAXH	0.351	33.68	69.6
MAXT	0.035	16.05	49.7
MAXW	0.359	35.99	69.6

LDA– linear discriminant analysis

The cut-off points of both studies were used in the calcined patella data set to verify their efficacy in the sample of the sub-collection of experimentally burnt individuals (Table 6). The cut-off points were also compared to the carbonized patellae, where males all had a correct classification, whereas the females yielded mixed results.

**Table 6:** Evaluation of the applicability of cut-off points established in two previous studies and the calcined patellae sample (*N* = 23, female *N* = 13, male *N* = 10) of the current study

Measurement	Cavazutti et al. [34]				Hlad et al. [35]			
	Cut-off point (mm)	Female (%)	Male (%)	Total (%)	Cut-off point (mm)	Female (%)	Male (%)	Total (%)
MAXH	35.68	76.9	50	65.2	36.30	76.9	65.2	65.2
MAXT	16.10	54.8	50	52.2	16.30	53.8	50	43.5
MAXW	36.61	76.9	69	69.7	38.10	76.9	60	69.7

## Discussion

### Sexual dimorphism evaluation in experimentally burnt patellae

The results obtained showed that in the studied sample of patellae experimentally burnt, at different temperatures and durations, sexual dimorphism is preserved to a certain degree, with the D-value lying between 0.220 and 0.453. This is in line with previous studies [34, 35], that have studied cremated patellae, or as another study [26] documented in other bones (humerus, femur, talus and calcaneus) from a Portuguese sample. The insights gained from this study may help researchers anticipate the condition of bones after prolonged exposure to fire, opening new opportunities for further studying. Additionally, this research aids in developing more accurate methods for interpreting burn patterns and estimating sex from burned remains.

Nevertheless, the results from the current study show that none of the evaluated measurements achieved the benchmark value of 80% or greater, which is commonly used to indicate the precision of a sex estimation method and values below this threshold usually are considered insufficient for practical applications [45, 46]. The values of correct sex classification in the current study are of 68.8–75%, which shows that although the accuracy percentage does not reach the standard minimum of 80%, the values are still significant given the alterations the patellae have undergone. These findings provide important insights into the morphological changes and preservation of sexually dimorphic traits in bones exposed to high temperatures. By documenting how burn-induced alterations affect traditional sex estimation methods, this research lays the groundwork for developing improved techniques and refining existing protocols. The burnt patellae sample have similar percentages to the correct classifications seen in non-burnt patellae of ISC/XXI, with values between 77.5% and 81.1% [18], and the accuracy of sex estimation remaining close to the radius and tarsal bones (synthesis Table 7 in Roggio and researchers [47]).

The results of this study could be biased by the relatively small sample size available. Nevertheless, the sub-collection of experimentally burnt skeletons used is unique and the only one of its kind in the world [38], and this is a pioneer study of the impact of thermal exposure to the biological sex estimation in patellae. Additionally, it is important to consider other factors that may affect the accuracy of sex estimation methods, such as a quite high average age at death (78.7 years). The decrease in bone mineral content, seen in older individuals, has most likely had an impact on the sexual dimorphism, as this phenomenon can have implications for the size and shape of bones [48, 49], especially in the spongy bone, which would affect the patella further. Furthermore, the different factors associated with the combustion of the patella could have influenced the original dimensions, such as heat induced shrinkage, which once evaluated in the same sample was higher for males. Although this is not impeding of sex estimating, as Thompson [50] and Gonçalves and colleagues [26] have stated for other bones.

The D-values achieved show that MAXT exhibits the lowest degree of sexual dimorphism (D-value of 0.220). This could have occurred due to the higher trabecular bone content in the anteroposterior view of the patella when compared to the other dimensions, as it tends to shrink more than compact bone [51–53]. MAXW has the highest D value (0.453) while MAXH has an intermediate D-value of 0.423, which indicates reasonable sexual dimorphism in these dimensions of burnt patellae. This means that males retain a bigger patella with the biggest differences more noticeable in height and width. Overall, the findings suggest that caution should be taken when interpreting D-values, especially when using small sample sizes. As expected, when compared to the unburnt left patellae the D-values of burnt samples are lower, with the biggest difference seen in the MAXW.

By using the discriminant function, it is possible to assign individuals to the most appropriate group based on their scores on the three measurements, which can lead to more effective decision-making [11, 17]. Thus, it is a good option as it allows for more precise and accurate classification of individuals based on multiple measurements [54], in this instance patellar maximum height, thickness and width. Additionally, this helps to identify patterns and relationships between variables that may not be evident when looking at them individually. Although the three measurements show a good sexual dimorphism individually and with their cut-off points, since the current study has been developed in an experimentally burnt collection with different degrees of burning, the equation with a more accurate sex estimation is the one where all three traits are employed.

### Comparison with two previous studies

When compared to the two studies [34, 35], the D-values are higher for the three measurements evaluated. Per this study, in the calcined sample, the MAXT of the patella shows to be the most dimorphic feature, contrasting with the previous research. This study’s results show that the D-values obtained for the maximum thickness, height and width of the patella are close to those found previously [35] (Table 5).

In the calcined patellae, the D-values follow a similar trend, and the area of non-overlap is lowered when only these are evaluated. The MAXW (0.359) is slightly higher than the MAXH (0.351), followed by MAXT, with a very low value of sexual dimorphism (0.035). This is congruent with the research done by the selected studies [34, 35], as MAXW shows the highest value of sexual dimorphism, accompanied by MAXH and then MAXT. Nonetheless, the patellae of the current study show lower values of sexual dimorphism, when compared to the two previous studies (Tables 4 and 5). The differences in biogeography and the average age at death are the most likely causes of these dissimilarities.

The findings of this study agree with those of Hlad and colleagues [35], who found that D-values for the three measurements were closer, ranging between 0.6 and 0.7, with MAXW having the highest value and MAXH having the lowest. Once again, the different values of sexual dimorphism found between the current study and that of Hlad et al. [35] can be explained by the smaller age range of our sample, as well as the different average ages at death. Hlad et al. [35] analysed individuals born in the 20th century with ages at death ranging from 32 to 101 years old (an average of 66.5 years at death). The burning protocols were different, cadavers were burned to complete calcination following crematorium rules of the USA, whereas the patellae in this study were subjected to burning in different temperatures and durations in a furnace. The comparison between the cut-off points of Hlad et al. [35] to the calcined patellae sample yielded similar results between MAXH (65.2%) and MAXW (69.7%), however, MAXT had a much lower correct classification result of 43.5%.

The lower sexual dimorphism found in the present study compared to what was noted by Cavazzuti et al. [34] could be explained by what was previously stated and the inferring of sex through gendered grave goods. Nevertheless, the authors pointed out a strong correlation between the materials and the osteological data, sexual identity is coincident with gender in most cases [34]. In this study the individuals lived in Portugal in the 20th -21st century, while the sample analysed by Cavazzuti et al. [34] represented individuals, from Italian sites dated between the Bronze and Iron Ages [55].

The variation in temperatures achieved during the burning process can also have implications for sexual dimorphism. The Italian sample concerns individuals cremated shortly after death on a pyre, where a multitude of variables occurs such as temperatures, the distance to the fire, whether it was a fresh corpse or already undergoing active decomposition or the availability of oxygen, which all will the dictate the cremation process. Thus, the process of combustion is very different from the current study since the experimentally burnt patellae were subjected to controlled burning.

When evaluating the percentages of correct classification and in contrast to the findings of the previously mentioned studies [34, 35], the current identified MAXH and MAXW as the two measurements with the highest discriminatory power (71.9% and 75.0%, respectively), after cross-validation. Although the correct classification percentage for MAXT was slightly lower (68.8%), the difference was small compared to the other measurements. Moreover, the combined accuracy rate of the measurements was reliable, with a 71.9% correct classification. Concerning only the calcined patellae, the accuracy rate of the measurements is significantly lower in MAXT (49.7%), whereas in MAXH and MAXW the correct classification percentage is only slightly lower (69.6%).

## Conclusion

Burnt bones undergo significant structural changes that make them significantly different from unburnt bones, as has been highlighted. Thus, the results obtained in this study cannot be directly extrapolated to unburnt patellae. Moreover, in the current study, the patella showed to be a good bone for sex estimation in burnt individuals, with special attention to patellar height and width, which is in accordance with previous studies. However, none of the values reached the minimum value of threshold value of 80%, thus it does not fulfil the necessary requirements to be considered in forensic studies.

Despite this, the findings contribute valuable data that enhance the understanding of sexual dimorphism in skeletal remains subjected to high temperatures. The methodology and results offer insights into how heat induced changes might affect morphological features used in sex estimation. These insights are important for developing more accurate techniques and refining existing methods, ultimately improving the reliability of sex estimation in forensic contexts. It can eventually be used as a complementary means, provided there is an estimate based on more precise methods. As the patellae used in this study had been experimentally burnt at different degrees i.e. different temperatures and burning durations, it can be of importance for sex estimation in forensic investigations, due to the variability seen in these cases. Controlled burns allow for a systematic and replicable approach to understanding how bones respond to specific temperature ranges and durations of heat exposure. This controlled environment helps in isolating variables and establishing baseline data, which can be used as a starting point for future studies analysing heat related bone shrinkage.

The cut-off points of two previous studies were compared to the calcined group of the experimentally burnt patellae and found that it has an accuracy of correct sex estimation of over 50% in all measurements, with the maximum value being 69.7% for the MAXW. Due to the low accuracy of sex estimation in these cut-off points, they should not be applied on modern Portuguese samples.

Furthermore, the analysis revealed that the measurements had a higher percentage of correct classification for females, with maximum width being the measurement that displayed the highest percentage of correct classification (75%). However, due to the limited available sample further studies should be employed to verify the reliability of this statement. Additionally, a sample with more heterogeneity of ages would be beneficial considering sexual dimorphism can reduce with an increase in age. Although not applicable to forensic casework, this research underscores the continuous need to refine methods, thereby enhancing the knowledge of forensic analyses when heat altered bone is present.
